# Efficacy and Safety of Tacrolimus Therapy for a Single Chinese Cohort With Very-Late-Onset Myasthenia Gravis

**DOI:** 10.3389/fneur.2022.843523

**Published:** 2022-03-30

**Authors:** Yiming Zheng, Xiaoqiu Yuan, Caifeng Zhang, Ran Liu, Haiqiang Jin, Hongjun Hao, Fan Li, Yawen Zhao, Yun Yuan, Zhaoxia Wang, Feng Gao

**Affiliations:** ^1^Department of Neurology, Peking University First Hospital, Beijing, China; ^2^North China University of Science and Technology Affiliated Hospital, Qinhuangdao, China

**Keywords:** myasthenia gravis, tacrolimus, late-onset, elderly, Wuzhi capsules, clinical efficacy

## Abstract

**Background and Purpose:**

Previous studies have found tacrolimus to be a favorable drug for treating different types of myasthenia gravis (MG), but few have focused on very-late-onset MG (VLOMG). This study evaluated the efficacy and safety of tacrolimus for VLOMG therapy.

**Methods:**

This was a retrospective single-center cohort study of 70 patients with VLOMG (onset ≥65 years) who visited Peking University First Hospital in 2019. Participants were divided into the tacrolimus (Tac) group and the control group based on tacrolimus usage. We further divided the Tac group into patients treated without corticosteroids and with corticosteroids. Sociodemographic features, clinical profiles, and outcomes were compared between different therapies and further analyzed by multivariate regression. Details of tacrolimus treatment, comorbidities, and adverse drug reactions (ADRs) were described.

**Results:**

Among 70 patients, the median (interquartile range) age at onset was 71 (68–77) years, and the follow-up duration was 27 (27-29) months. Most patients were types I (28%) and III (40%) according to the MG Foundation of America (MGFA) classification. In the Tac group, tacrolimus treatment was maintained for 36 (27-38) months. The dosage at the final evaluation was 1.0 (1.0–1.75) mg/day, and the last blood concentration test was 4.25 (2.85–5.7) ng/ml. A total of 43% reached remission, and 37% improved based on MGFA postintervention status (MGFA-PIS). For the 9 patients, newly diagnosed at enrollment within this group, MG activities of daily living (MG-ADL) decreased significantly from 3 (2-5) to 2 (1-2) (*p* = 0.041). Regarding the 13 patients, coadministering Wuzhi capsules the tacrolimus concentration increased from 2.75 (1.4–3.8) ng/ml to 5.95 (5.1–7.0) ng/ml (*p* = 0.012). No significant differences in outcomes were observed between tacrolimus treatment without and with corticosteroids or between the Tac group and the control group. A total of 93% had at least one comorbidity. ADRs related to tacrolimus emerged in 25% (9/36) of patients, most of which were not serious and reversible.

**Conclusions:**

Tacrolimus is effective and safe in treating VLOMG. Tacrolimus monotherapy without corticosteroids can be used as an initial and maintenance treatment for VLOMG. Wuzhi capsules work well in elevating tacrolimus concentrations in this population.

## Introduction

Myasthenia gravis (MG) is a neuromuscular junction disorder caused by autoantibodies against acetylcholine receptors (AChR) on the postsynaptic membrane or their adjacent proteins. Patients typically suffer from fluctuating fatigability of skeletal muscles, usually starting with ptosis or diplopia, and in severe cases progressing to respiratory muscle paralysis (1, 2). The recent studies suggested dividing the MG population into three groups based on the onset age using cutoffs of 50 and 65 years. Very-late-onset MG (VLOMG) refers to individuals developing symptoms at ≥ 65 years (3–5), which accounts for the largest proportion of all the MG age groups at present and exhibits an increasing incidence over recent years (3, 6, 7). VLOMG features vs. younger patients of more severe onset, higher risk of exacerbation, fewer medication requirements, better long-term outcomes, and more comorbidities have been reported, indicating potential divergence in treatment selections (3, 5, 8). In addition, age-related dysregulation of the immune system and comorbidities entangle considerations for management. The current treatments for VLOMG mainly work on enhancing hindered transmission (cholinesterase inhibitors) and suppressing overactivated humoral immunity (corticosteroids, immunosuppressors, or monoclonal antibodies). Rapid-acting treatments used in crisis include plasmapheresis and intravenous immunoglobulin (2, 9). Surgical removal of the thymus is less considered for the elderly regarding their comparatively fragile physical conditions and intolerance to surgery (1).

Tacrolimus could be a promising immunosuppressor in managing VLOMG owing to its rapid and long-term efficacy with relatively safe profiles, especially slight nephrotoxicity, which prominently benefits elderly patients (9–12). This agent inhibits T-cell activation by suppressing calcineurin and additionally strengthens muscle contractility by improving ryanodine receptor function (13). Several studies have confirmed the steroid-sparing and symptom-improving effects of tacrolimus in different types of MG (13–16). It is also necessary to clarify medication details for VLOMG, but such research is still lacking. Currently, the auxiliary roles of Chinese medicines in treating autoimmune diseases and preventing complications after transplantation are rising. Notably, Wuzhi capsules (WZC), one preparation of the ethanol extract of Wuweizi or *Schisandra sphenanthera*, have been reported to strongly improve the tacrolimus concentration (17–19). However, no study has focused on WZC usage in the older MG patients. Therefore, we investigated the efficacy and safety of tacrolimus in treating VLOMG and coadministration of WZC in this population and tried to perceive patients' feelings during treatment using patient-reported outcomes and scales of life quality in real-world settings.

## Materials and Methods

### Design and Patients

This study was approved by the Institutional Review Board and Ethics Committee at Peking University First Hospital and was conducted following the ethical standards of the Helsinki Declaration. Informed consent was obtained from each patient included.

**Figure 1:**
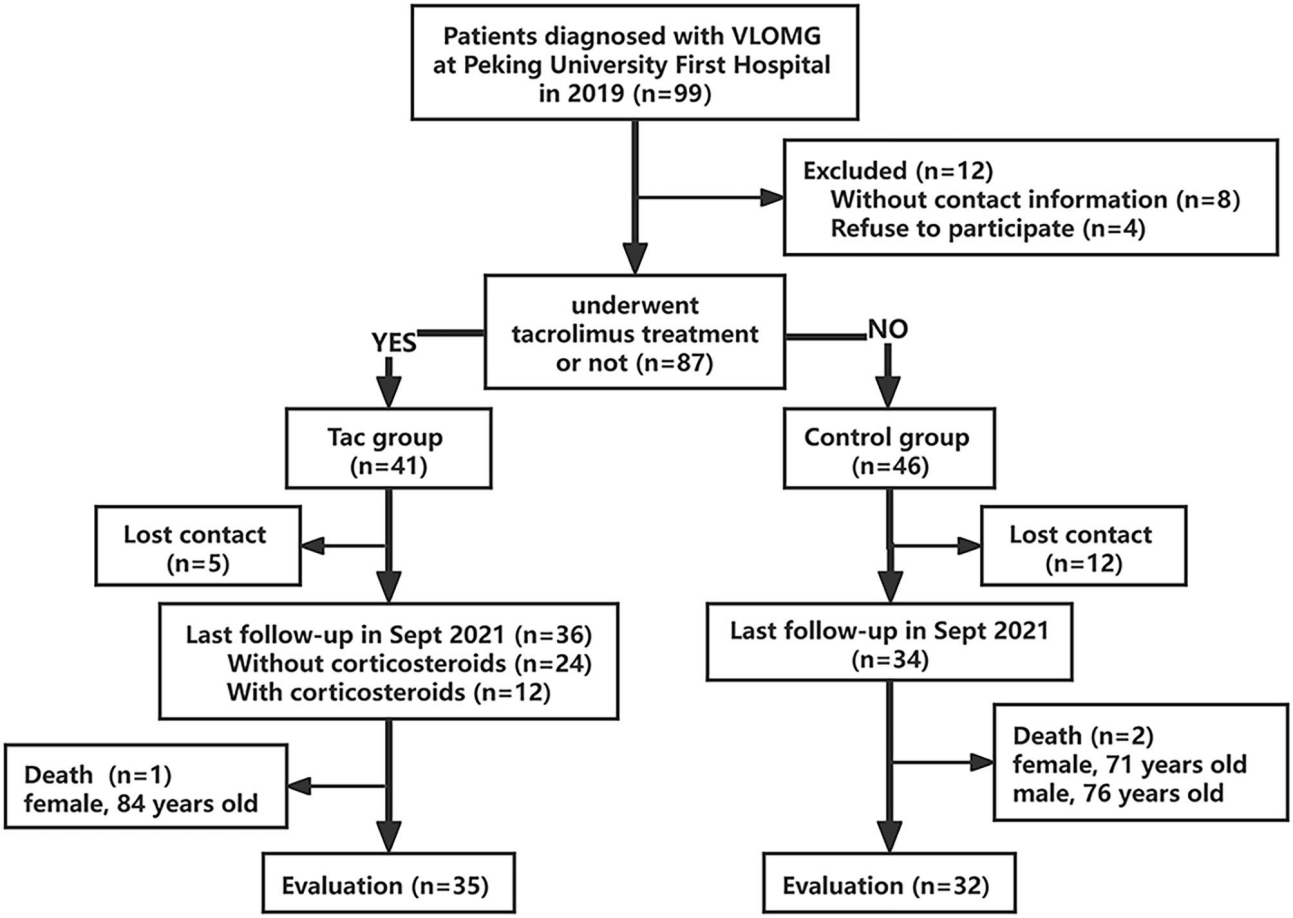
Flowchart of the participants included in this study. VLOMG, very-late-onset myasthenia gravis.

The retrospective single-center cohort study was performed under real-world settings in patients with very-late-onset MG (age at onset ≥ 65 years old). Participants included all individuals who visited our institution between January 15, 2019 and December 26, 2019, had a confirmed diagnosis of MG, and first developed symptoms at or after 65 years of age. Diagnoses of MG were made following the guidelines of the Association of British Neurologists based on the typical manifestations of fluctuating muscle weakness and diagnostic test results of antibody positivity or neurophysiological examination positivity (20). Those without contact information or those who refused to participate were excluded. Patients were divided into two groups as follows: the Tac group, participants who had been treated with tacrolimus longer than 3 months [tacrolimus took effect after 3 months of continuous medication in most patients according to previous studies (12, 21)]. Control group, those who had never used tacrolimus or those treated with this drug for less than 3 months. The Tac group was further divided into the non-corticosteroids (those who had never taken corticosteroids) and the corticosteroids group (those who had been treated by corticosteroids). Lost contact was defined as patients who did not answer the phone at least 3 times in different periods of the day or those who left the wrong numbers or empty numbers in records. The flowchart of patient selection in the study is presented in Figure 1.

### Covariates

Sociodemographic features (sex, age at MG onset, age at the last follow-up, resident area, nationality, marital status, occupation, and educational attainment) were obtained from retrospective analyses of the hospital records. Details of treatments from the patients' medical records and their report at the last follow-up included tacrolimus, cholinesterase inhibitors, corticosteroids, WZC, other immunosuppressors (including azathioprine, cyclosporin A, mycophenolate mofetil, methotrexate, cyclophosphamide, and rituximab), other Chinese medicines (except for the natural product and extracts of Wuweizi or *Schisandra sphenanthera*), concomitant drugs (drugs taken to treat concomitant diseases), intravenous immunoglobulin (IVIg), and plasmapheresis. Additional data on tacrolimus and WZC were collected: age of starting tacrolimus, the dose of tacrolimus at the final evaluation (for those with discontinuation, we collected the dosage before withdrawal documented in the medical records), last tacrolimus concentration, duration of continuous medication (from starting tacrolimus to the last follow-up or discontinuation), WZC usage, and tacrolimus concentration before and after taking WZC.

Information on comorbidities was obtained from medical records, digital questionnaires, and the follow-up telephone interviews as a supplement. This part of the interviews included several closed questions and one open question allowing respondents to add information. Comorbidities being queried included hypertension, coronary diseases, arrhythmia, valvular diseases, peripheral vascular diseases, cerebrovascular diseases, other neurological diseases, diabetes, thyroid diseases, dyslipidemia, hyperuricemia, chronic lung disease, digestive diseases, hepatobiliary disease, kidney diseases, eye diseases, skin diseases, osteoporosis or fracture, depression, infections, hematopoietic diseases, benign prostatic hyperplasia, gynecological diseases, and malignant solid tumor. Here, we used the number of comorbidities to quantify the burden of concomitant diseases. Other collected clinical characteristics included the first visit or revisit at enrollment, inpatient or outpatient visit, length of follow-up, anti-AChR antibody positivity, thymic pathology, MG subtype according to MG Foundation of America (MGFA) classification, and baseline MG activities of daily living (MG-ADL). Anti-AChR antibodies were measured by radioimmunoassay in the neuroimmunology laboratory of Peking University First Hospital. Thymic hyperplasia or thymoma was diagnosed based on the CT scan results. MGFA type and MG-ADL were assessed by the attending neurologists.

### Therapeutic Regimens

Treatment was tailored to the individual patient following guidelines, the experience of the clinicians, and patient wishes. For mild patients with ocular MG, cholinesterase inhibitors were prescribed; a single 60 mg of cholinesterase inhibitors (pyridostigmine bromide) was administered when symptoms occurred (≦480 mg/day). Immunosuppression was added when inadequate response occurred or in patients with generalized MG. Prednisone or equivalent methylprednisolone was started at 20 mg/day and increased by 10 mg every 5 to 7 days to the daily target of 0.5–1.0 mg/kg (≦100 mg/day); after 6–8 weeks of maintenance, the dose of steroids was gradually reduced to the lowest effective dose. For those who did not require rapid improvement (such as myasthenic crisis or life being severely affected) or who could not tolerate the side effects of steroids, tacrolimus, and other immunosuppressors were considered. In the Tac group, tacrolimus was administered twice a day at an initial dose of 1.0 mg/day, following the physicians' advice. Dosage was adjusted based on the individual's disease condition, tacrolimus concentration, tests of hematology, and biochemistry. For those who could not achieve adequate concentration (4.8–9.0 ng/ml) (22, 23) by adjusting doses, two tablets of WZC were taken with tacrolimus every time. In the control group, stable doses of other immunosuppressors were as follows: azathioprine 2–3 mg/kg/day, cyclosporin A 2–4 mg/kg/day, mycophenolate mofetil 1.0–1.5 g/day, methotrexate 20 mg/week (with folic acid 1 mg/day), and cyclophosphamide 100 mg/day. Rituximab was administered 375–750 mg/m^2^ once every 2 weeks. IVIg (400 mg/kg/day for 5 days) or plasmapheresis (each exchange of 1.0–1.5 plasma volumes and 3–6 times within 10–14 days) were performed in life-threatening cases. Other Chinese medicines and concomitant drugs were applied following doctors of other specialties.

### Outcome Measurements and Follow-Up

To evaluate the efficacy of tacrolimus and its impacts on quality-of-life, MGFA postintervention status (MGFA-PIS), MG-ADL scale, relapse (s) or aggravation (s), revised 15-item MG Quality-of-Life scale (MG-QOL-15R), simple single question (SSQ), and five-level EuroQol five-dimensional questionnaire (EQ-5D-5L) were assessed based on semistructured telephone interviews at the last follow-up in September 2021. If patients were not able to answer the questions, close family members were interviewed instead. The primary outcome was MGFA-PIS, the secondary outcome consisting of the other measurements mentioned above. Details of adverse drug reactions (ADRs) were extracted from the interviews.

The MGFA-PIS served as a four-categorical variable in this study: remission (chronic stable remission or minimal manifestations or pharmacological remission), improved, unchanged, and worse or exacerbation (24). In the Tac group, patients were additionally divided into the effective and ineffective groups according to whether MGFA-PIS remission/improvement was achieved. A higher MG-ADL score (range: 0–24) indicates greater MG-related disability (24). The change of MG-ADL was calculated by subtracting the baseline score from the follow-up score. Relapse(s) or aggravation(s) was evaluated by the patients' description after being asked “Have you ever relapsed or aggravated since (the date at enrollment)”. Relapse was defined as the reappearance of any symptoms that lasted more than 24 h, and aggravation was defined according to MGFA-PIS criteria of worse or exacerbation. A higher MG-QOL-15R score (range: 0–30) reflects worse physical and psychological functionality (25). The eighth question focusing on work skills might not fit in our patients since the majority were retired. We jumped over it and used 0 as the unified score for this question. The SSQ is a simple and validated question of “What percentage of normal do you feel regarding your MG” (26). Most patients could only name a range, so substitutions (1–10% = 1, 11–20% = 2, and so on) were adopted. EQ-5D-5L health utility values (hereafter referred to as health value) were calculated according to an 8-parameter multiplicative model that performed well in the Chinese individuals (27).

### Statistical Analyses

Analyses were performed using Stata version 12.0 (StataCorp LLC). *P* < 0.05 (2-sided) was considered statistically significant. Continuous variables were expressed as medians and interquartile ranges (IQRs) according to a skewed distribution, while categorical variables were described by percentages. Information on patients who lost contact [20% (17/87)] was not collected. Comorbidities, outcome measurements, and ADRs of the deceased [4% (3/70)] were not available. Items with incomplete data (sociodemographic characteristics and anti-AChR antibody positivity) are presented as *n*/total (%). All these missing data were not involved in the statistical analyses.

Confounding factor adjustment was conducted on covariates with *p* < 0.05 in univariable comparisons using multiple linear regression (continuous outcomes) and multiple modified Poisson regression (categorical outcomes). Modified Poisson regression (Poisson regression with robust estimation of variance) instead of logistic regression was applied in this study to allow a more accurate estimation of rate ratio (RR) when the rare event assumption was violated (28).

To evaluate the efficacy of tacrolimus, univariable comparisons of covariates and outcomes were conducted between the Tac group and the control group using the Mann–Whitney U test for continuous variables and the chi-squared test for categorical variables. Covariates with *p* < 0.05 were then included in multivariate regression models. The presence of multicollinearity was evaluated by variance inflation factor estimates. Similar analyses were performed in two subgroups: “First visit,” 19 patients who first visited our clinic and were newly diagnosed with MG at enrollment (Tac: 9, control: 10). “MGFA>1,” 50 patients whose MGFA type was higher than I (Tac: 31, control: 19). To evaluate the efficacy of tacrolimus monotherapy, univariable comparisons using the Fisher exact test for categorical variables and Mann-Whitney U test for continuous variables were conducted between the corticosteroids and non-corticosteroids groups. And, we applied similar comparisons between ocular and generalized MG patients in the Tac group to assess the efficacy of tacrolimus in different MG types. To explore potential factors relevant to outcomes, univariable comparisons of covariates were performed between the effective and ineffective groups. Paired *t*-test was utilized to compare twice-repeated measurements.

## Results

### Patient Characteristics

**Figure 2:**
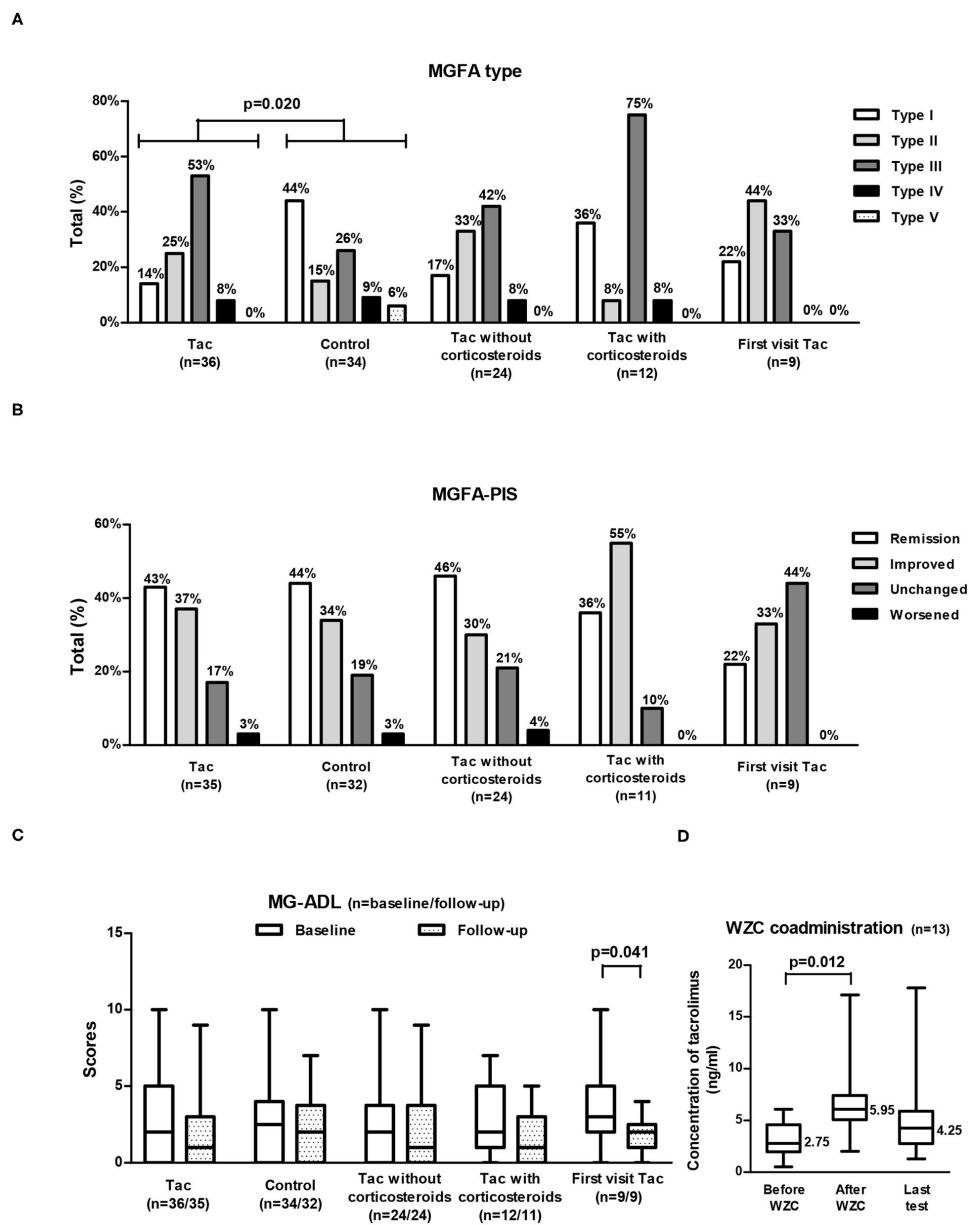
MGFA type **(A)**, MGFA-PIS at follow-up **(B)**, and MG-ADL **(C)** in different groups of VLOMG patients and changes in tacrolimus concentrations with WZC coadministration **(D)**. MGFA type and baseline MG-ADL shared the same sample size of each group [as displayed in **(A,C)**]; MGFA-PIS and MG-ADL at follow-up were not available for the three deceased patients and they also shared the sample size presented in **(B,C)**. A total of 13 patients coadministrated WZC **(D)**. VLOMG, very-late-onset myasthenia gravis; Tac, the tacrolimus (Tac) group; control, the control group; First visit Tac, 9 newly diagnosed patients in the Tac group; MGFA, Myasthenia Gravis Foundation of America; MGFA-PIS, MGFA Post-intervention Status; MG-ADL, MG Activities of Daily Living; WZC, Wuzhi capsules. The chi-square tests were performed to compare the MGFA type or the MGFA-PIS. The Mann–Whitney U tests were utilized to compare the MG-ADL scores of different groups. For comparisons of the MG-ADL scores at baseline and follow-up or tacrolimus concentrations before and after coadministrating WZC, paired *t*-tests were conducted. Box-and-whisker plots in **(C,D)** show the medians, interquartile ranges, and min to max ranges. Significant *p* values are presented.

A total of 70 patients with VLOMG (30 females, 40 males) were eligible for this study, comprising 36 patients in the Tac group and 34 in the control group. Demographical and clinical characteristics are summarized in Table 1. The median (interquartile range) age was 71 (68–77) years at disease onset and 77 (72–81) years at the last evaluation. The median duration of follow-up was 27 (27–29) months. At the time of enrollment, the median MG–ADL score was 2 (0–4); 19 patients (27%) were newly diagnosed with MG, and 15 (21%) patients were hospitalized for MG. Distributions of MGFA classification in different groups are presented in Figure 2A. A total of 16 patients (23%) had thymoma or thymic hyperplasia. Antibodies were tested in 62 patients (89%), and 48 (79%) were anti-AChR antibody positive. The main treatments except for tacrolimus and WZC (these two are summarized in Table 2) are listed in Table 1.

**Table 1:** Demographical and clinical characteristics of VLOMG patients in the whole cohort and first visit subgroup.

**Variables**	**Whole (*****n*** **=** **70)**				**First visit (*****n*** **=** **19)**			
	**Control group** **(*n* = 34)**	**Tac group (*n* = 36)**	**Total** **(*n* = 70)**	***P* value^**a**^**	**Control group** **(*n* = 10)**	**Tac group** **(*n* = 9)**	**Total** **(*n* = 19)**	***P* value**
**Demographic characteristics**								
Sex				0.084				0.876
Male	23 (68%)	17 (47%)	40 (57%)		7 (70%)	6 (67%)	13 (68%)	
Female	11 (32%)	19 (53%)	30 (43%)		3 (30%)	3 (33%)	6 (32%)	
Age at onset, median (IQR), y	70.5 (67–77)	72 (68.5–78)	71 (68–77)	0.491	70 (68–79)	73 (70–76)	70 (68–78)	0.742
Inpatient at enrollment	10 (30%)	5 (14%)	15 (21%)	0.114	5 (50%)	2 (22%)	7 (37%)	0.210
Follow-up time, median (IQR), m	27 (26–29)	28 (27–29)	27 (27–29)	0.256	26.5 (23–29)	28 (25–29)	27 (25–29)	0.711
**Clinical profiles**								
Anti-AChR antibody positive	22/31 (71%)^b^	26/31 (84%)	48/62 (79%)	0.224	7 (70%)	7/8 (88%)	14/18 (78%)	0.375
Thymoma or thymic hyperplasia	6 (18%)	10 (28%)	16 (23%)	0.313	1 (10%)	3 (33%)	4 (21%)	0.213
MGFA type				0.020^*^				0.073
I	15 (44%)	5 (14%)	20 (28%)		6 (60%)	2 (22%)	8 (42%)	
IIa/IIb	5 (15%)	9 (25%)	14 (20%)		0 (0%)	4 (44%)	4 (21%)	
IIIa/IIIb	9 (26%)	19 (53%)	28 (40%)		3 (30%)	3 (33%)	6 (32%)	
IVa/IVb	3 (9%)	3 (8%)	6 (9%)		0 (0%)	0 (0%)	0 (0%)	
V	2 (6%)	0 (0%)	2 (3%)		1 (10%)	0 (0%)	1 (5%)	
MG-ADL at enrollment, median (IQR)	2.5 (0–4)	2 (0–5)	2 (0–4)	0.809	3 (1–3)	3 (2–5)	3 (2–3)	0.365
**Details of treatments**								
Corticosteroids				0.015^*^				0.364
Never use	16 (47%)	24 (67%)	40 (57%)		7 (70%)	8 (89%)	15 (79%)	
Using	14 (41%)	4 (11%)	18 (26%)		2 (20%)	0 (0%)	2 (11%)	
Discontinuation	4 (12%)	8 (22%)	12 (17%)		1 (10%)	1 (11%)	2 (11%)	
Other immunosuppressors^c^				0.038^*^				0.073
Never use	23 (68%)	33 (92%)	56 (80%)		7 (70%)	9 (100%)	16 (84%)	
Using	9 (26%)	2 (6%)	11 (16%)		3 (30%)	0 (0%)	3 (16%)	
Discontinuation	2 (6%)	1 (3%)	3 (4%)		0 (0%)	0 (0%)	0 (0%)	
Cholinesterase inhibitors				0.488				0.161
Never use	9 (26%)	8 (22%)	17 (24%)		2 (20%)	0 (0%)	2 (11%)	
Using	18 (53%)	16 (44%)	34 (49%)		5 (50%)	8 (89%)	13 (68%)	
Discontinuation	7 (21%)	12 (33%)	19 (27%)		3 (30%)	1 (11%)	4 (21%)	
IVIg	8 (24%)	10 (28%)	18 (26%)	0.684	2 (20%)	2 (22%)	4 (21%)	0.906
Plasmapheresis	3 (9%)	0 (0%)	3 (4%)	0.069	0 (0%)	0 (0%)	0 (0%)	——
Other Chinese medicines^d^	9 (26%)	9 (25%)	18 (26%)	0.888	1 (10%)	1 (11%)	2 (11%)	0.937
Concomitant drug(s)^e^	27 (80%)	27 (75%)	54 (77%)	0.660	8 (80%)	8 (89%)	16 (84%)	0.596
**Comorbidities** ^**f**^	**(*****n*** **=** **32)**	**(*****n*** **=** **35)**	**(*****n*** **=** **67)**		**(*****n*** **=** **9)**	**(*****n*** **=** **9)**	**(*****n*** **=** **18)**	
Without comorbidity	3 (9%)	2 (6%)	5 (7%)	0.569	0 (0%)	1 (11%)	1 (6%)	0.303
Number of comorbidities, median (IQR)	3 (2–5.5)	2 (2–4)	3 (2–4)	0.178	4 (2–5)	2 (1–3)	3 (2–5)	0.194

* *Covariates with a p < 0.05 were included in multiple regression analysis*.

a *Compared the Tac group with the Control group*.

b *Incomplete data were described by n/total (%)*.

c *Included 8 methotrexate, 3 mycophenolate mofetil, 3 rituximab, 2 azathioprine, 1 cyclophosphamide, and 1 cyclosporin A*.

d *Except for the natural product and extracts of Wuweizi or Schisandra sphenanthera*.

e *Drugs are taken to treat concomitant diseases*.

f *Information on comorbidities was not available for the three deceased patients*.

*VLOMG, very-late-onset myasthenia gravis; Whole, 67 patients completing the last follow-up except 3 deceased; First visit, 19 patients who first visited our institution and were newly diagnosed with MG at enrollment; IQR, interquartile range; AChR, acetylcholine receptor; MG, Myasthenia Gravis; MGFA, Myasthenia Gravis Foundation of America; IVIg, intravenous immunoglobulin*.

**Table 2:** Clinical profiles and outcome measurements of VLOMG patients in the Tac group.

**Variables**	**Non-corticosteroids group (*n* = 24)**	**Corticosteroids group (*n* = 12)**	**Total (*n* = 36)**	***P* value**
Sex				0.302
Male	13 (54%)	4 (33%)	17 (47%)	
Female	11 (46%)	8 (67%)	19 (53%)	
**Clinical profiles**				
Anti-AChR antibody positive	17/21 (81%)	9/10 (90%)	26/31 (84%)	1.000
Thymoma or thymic hyperplasia	6 (25%)	4 (33%)	10 (28%)	0.700
MGFA type				0.224
I	4 (17%)	1 (8%)	5 (14%)	
IIa/IIb	8 (33%)	1 (8%)	9 (25%)	
IIIa/IIIb	10 (42%)	9 (75%)	19 (53%)	
IVa/IVb	2 (8%)	1 (8%)	3 (8%)	
V	0 (0%)	0 (0%)	0 (0%)	
MG-ADL at enrollment, median (IQR)	2 (0–3.5)	2 (1–5)	2 (0–5)	0.745
**Details of treatments**				
Age of starting tacrolimus, median (IQR), y	76 (69–80)	74 (70–77)	74 (69–79)	0.638
Duration of MG at tacrolimus initiation, median (IQR), m	4.5 (1–17.5)	16.5 (8–23.5)	8 (2–22)	0.060
Duration from starting tacrolimus to the last follow-up or withdrawal, median (IQR), m	37 (27.5–55)	36 (21–50)	36 (27–52)	0.467
Dose of tacrolimus at the final evaluation, median (IQR), mg/day	1.0 (1.0–1.75)	1.25 (1.0–1.75)	1.0 (1.0–1.75)	0.817
The last tacrolimus concentration, median (IQR), ng/ml	4.4 (2.7–6.7)	3.9 (3.1–5.0)	4.25 (2.85–5.7)	0.630
Wuzhi capsules	9 (38%)	4 (33%)	13 (36%)	1.000
Tacrolimus concentration before taking Wuzhi capsules, median (IQR), ng/ml	2.7 (1.1–3.8)	2.8 (1.4–6.1)	2.75 (1.4–3.8)	0.569
Tacrolimus concentration after taking Wuzhi capsules, median (IQR), ng/ml	6.4 (5.1–7.0)	4.45 (2.55–6.6)	5.95 (5.1–7.0)	0.285
Change in tacrolimus concentration, median (IQR), ng/ml	4.1 (2.9–5.6)	1.3 (0.6–3.0)	3.0 (2.4–4.5)	0.071
Tacrolimus discontinuation due to the already undercontrolled symptoms^a^	5 (21%)	4 (33%)	9 (25%)	0.443
Patient-reported ADRs caused by tacrolimus	5/24 (21%)	3/11 (27%)	8/35 (23%)	1.000
Other immunosuppressors^b^	1 (4%)	2 (17%)	3 (8%)	0.253
Cholinesterase inhibitors				0.736
Never use	6 (25%)	2 (17%)	8 (22%)	
Using	11 (46%)	5 (42%)	16 (44%)	
Discontinuation	7 (29%)	5 (42%)	12 (33%)	
IVIg	4 (17%)	6 (50%)	10 (28%)	0.053
Other Chinese medicines	6 (25%)	3 (25%)	9 (25%)	1.000
Concomitant drug(s)	17 (71%)	10 (83%)	27 (75%)	0.685
**Comorbidities** ^**c**^	**(*****n*** **=** **24)**	**(*****n*** **=** **11)**	**(*****n*** **=** **35)**	
Without comorbidity	1 (4%)	1 (9%)	2 (6%)	0.536
Number of comorbidities, median (IQR)	2 (1.5–4)	2 (1–4)	2 (1–4)	0.715
**Outcome measurements** ^**c**^	**(*****n*** **=** **24)**	**(*****n*** **=** **11)**	**(*****n*** **=** **35)**	
MGFA-PIS				0.578
Remission	11 (46%)	4 (36%)	15 (43%)	
Improved	7 (30%)	6 (55%)	13 (37%)	
Unchanged	5 (21%)	1 (10%)	6 (17%)	
Worsened	1 (4%)	0 (0%)	1 (3%)	
MG-ADL, median (IQR)	1 (0–3.5)	1 (0–3)	1 (0–3)	0.756
Change of MG-ADL^d^, median (IQR)	0 ([−1]−2.5)	1 (0–2)	0 ([−1]−2)	0.614
Have relapsed or aggravated^e^	7 (29%)	8 (73%)	15 (43%)	0.027^*^
MG-QOL-15R, median (IQR)	1 (0–7)	2 (0–7)	1 (0–7)	0.900
SSQ, median (IQR)	8 (6.5–9)	8 (7–9)	8 (7–9)	0.454
EQ-5D-5L health value, median (IQR)	0.91 (0.71–1.00)	0.90 (0.77–1.00)	0.90 (0.74–1.00)	0.730

a *Symptoms can be controlled with only cholinesterase inhibitors according to the physicians' orders*.

b *Included 1 mycophenolate mofetil, 1 rituximab, and 1 cyclophosphamide*.

c *A female patient in the Tac group died during follow-up. Information on her comorbidities, ADRs, and outcome measurements were not available. Of the 35 patients completing the last follow-up, 17 (49%) answered in person, 13 (54%) answered in the corticosteroids group, and 4 (36%) answered in the non-corticosteroids group (p = 0.328, no significant differences)*.

d *Subtract MG-ADL score at enrollment from the score at the last follow-up*.

e *This item was evaluated by the patients' description after being asked “Have you ever relapsed or aggravated since (the date at enrollment)”*.

*AChR, acetylcholine receptor; MG, Myasthenia Gravis; MGFA, Myasthenia Gravis Foundation of America; IQR, interquartile range; IVIg, intravenous immunoglobulin; MG-ADL, MG Activities of Daily Living; IQR, interquartile range; EQ-5D-5L, five-level EuroQol five-dimensional questionnaire; MG-QOL-15R, revised 15-item Myasthenia Gravis Quality of Life scale; SSQ, simple single question; MGFA-PIS, MGFA Post-intervention Status*.

* *Statistically significant*.

Three subjects died during follow-up: a female (Tac group, 84 years) died from cerebral infarction together with abundant comorbidities (lung infections, coronary artery disease, Hashimoto”s thyroiditis, peripheral neuropathy, gastroesophageal reflux, atherosclerosis, and hyperuricemia). Another female patient (control group, 71 years) died from an attack of myasthenic crisis at home, and a male patient (control group, 76 years) died from tracheotomy-related death with myasthenic crisis, unresponsive wakefulness syndrome, and aspiration pneumonia. Of the 67 patients completing the final evaluation, 62 (93%) had at least one comorbidity, and 54 (77%) were taking concomitant drugs other than treating MG. The median number of comorbidities was 3 (2–4), and the three most common comorbidities were hypertension [74% (50/67)], diabetes [34% (23/67)], and dyslipidemia [24% (16/67)]. Others with incidences higher than 10% included digestive system diseases [18% (12/67)], coronary heart diseases [16% (11/67)], osteoporosis or fracture [16% (11/67)], eye diseases [16% (11/67)], other neurological diseases except for MG and cerebrovascular diseases [15% (10/67)], benign prostatic hyperplasia [13% (5/39)], liver diseases [12% (8/67)] and gynecological diseases [11% (3/28)].

### Clinical Response to Tacrolimus Treatment in VLOMG

A total of 36 VLOMG patients had been treated with tacrolimus for more than 3 months, 9 of whom were newly diagnosed at enrollment. Clinical profiles and outcome measurements of the Tac group are summarized in Table 2, while outcomes of the first visit patients treated with tacrolimus (first visit Tac) are presented in Table 3. Of the 36 subjects, the median duration of MG at tacrolimus initiation was 8 (2–22) months. Age at initiation was 74 (69–79) years. A total of 23 (65%) had started before enrollment for 22 (15–32) months (minimum 5 months). By the last follow-up or discontinuation, tacrolimus treatment had lasted for 36 (27–52) months. The dosage at the final evaluation was 1.0 (1.0–1.75) mg/day, and the last blood concentration test was 4.25 (2.85–5.7) ng/ml. Nine (25%) discontinued tacrolimus since their symptoms only needed to be controlled with pyridostigmine according to the physicians' orders.

**Table 3:** Outcome measurements of VLOMG patients in the whole cohort and subgroups^a^.

**Outcome**	**Whole (*****n*** **=** **67)**				**First visit (*****n*** **=** **18)**			
	**Control group (*n* = 32)**	**Tac group (*n* = 35)**	**Total (*n* = 67)**	***P* value**	**Control group (*n* = 9)**	**Tac group (*n* = 9)**	**Total (*n* = 18)**	***P* value**
MGFA-PIS				0.995				0.214
Remission	14 (44%)	15 (43%)	29 (43%)		5 (56%)	2 (22%)	7 (39%)	
Improved	11 (34%)	13 (37%)	24 (36%)		3 (33%)	3 (33%)	6 (33%)	
Unchanged	6 (19%)	6 (17%)	12 (18%)		1 (11%)	4 (44%)	5 (28%)	
Worsened	1 (3%)	1 (3%)	2 (3%)		0 (0%)	0 (0%)	0 (0%)	
MG-ADL, median (IQR)	2 (0–3.5)	1 (0–3)	1 (0–3)	0.690	1 (0–2)	2 (1–2)	1.5 (0–2)	0.272
Change of MG-ADL^b^, median (IQR)	−1 ([−2]−1)	0 ([−2]−1)	−1 ([−2]−1)	0.944	1 (1–2)	1 (0–3)	1 (0–3)	0.755
Have relapsed or aggravated^c^	17 (53%)	15 (43%)	32 (48%)	0.401	3 (33%)	3 (33%)	6 (33%)	1.000
MG crisis attack during follow-up	3/34 (9%)	4/36 (11%)	7/70 (10%)	0.750	1/10 (10%)	0/9 (0%)	1/19 (5%)	0.330
MG-QOL-15R, median (IQR)	2 (0–6)	1 (0–7)	1 (0–6)	0.737	3 (0–7)	1 (1–2)	1 (0–6)	0.752
SSQ, median (IQR)	7.5 (6–9)	8 (7–9)	8 (6–9)	0.400	8 (7–10)	9 (8–9)	8.5 (7–9)	0.964
EQ-5D-5L health value, median (IQR)	0.88 (0.80–0.96)	0.90 (0.74–1.00)	0.88 (0.77–1.00)	0.790	0.83 (0.83–0.91)	1.00 (0.88–1.00)	0.89 (0.83–1.00)	0.079

a *Outcome measurements of the three deceased patients were not available. Of the 67 patients completing the last follow-up, 36 (54%) answered in person, 19 (60%) in the control group, and 17 (49%) in the Tac group (p = 0.376). There were also no significant differences in respondents in the two subgroups*.

b *Subtract MG-ADL score at enrollment from the score at the last follow-up*.

c *This item was evaluated by the patients' description after being asked “Have you ever relapsed or aggravated since (the date at enrollment).” Whole, 67 patients completed the last follow-up except 3 deceased; First visit, 19 patients who first visited our institution and were newly diagnosed with MG at enrollment; MGFA > 1, 50 patients whose MGFA type was higher than I; MG-ADL, MG Activities of Daily Living; IQR, interquartile range; EQ-5D-5L, five-level EuroQol five-dimensional questionnaire; MG-QOL-15R, revised 15-item Myasthenia Gravis Quality of Life scale; SSQ, simple single question; MGFA-PIS, MGFA Post-intervention Status*.

Concerning MGFA-PIS (Figure 2B), 43% of the Tac group achieved remission (first visit Tac: 22%), and 37% had improved (first visit Tac: 33%). The MG-ADL score of the entire Tac group remained stable during follow–up. However, among the 9 newly diagnosed patients, the MG–ADL score decreased significantly from 3 (2–5) to 2 (1–2) (*p* = 0.041, paired *t*-test) (Figure 2C). A total of 15 patients (43%) recalled relapse(s) or aggravation(s) [first visit Tac: 3 (33%)] after initiation of tacrolimus. Out of the 4 newly diagnosed patients with “unchanged” status, two were MGFA type I, one was MGFA type II, and the other was MGFA type III with five comorbidities (hypertension, cranial trauma, rectal polyps, hypothyroidism, and atherosclerosis). We did not discover evident differences in comparing the outcomes between generalized MG (*n* = 30) and ocular MG patients (*n* = 5). In the exploration of factors relevant to outcomes (Table 4), no significant differences were found in the clinical characteristics and medication details between the effective (*n* = 28) and ineffective groups (*n* = 7).

**Table 4:** Characteristics of VLOMG patients in the Tac group according to clinical outcome.

**Variables**	**Ineffective group (*n* = 7)**	**Effective group (*n* = 7)**	**Total (*n* = 35)**	***P* value**
Sex				0.612
Male	4 (57%)	13 (46%)	17 (49%)	
Female	3 (43%)	15 (54%)	18 (51%)	
**Clinical profiles**				
Anti-AChR antibody positive	7/7 (100%)	19/23 (83%)	26/30 (87%)	0.236
Thymoma or thymic hyperplasia	1 (14%)	9 (32%)	10 (29%)	0.350
MGFA type				0.841
I	1 (14%)	4 (14%)	5 (14%)	
IIa/IIb	1 (14%)	8 (29%)	9 (26%)	
IIIa/IIIb	4 (57%)	14 (50%)	18 (51%)	
IVa/IVb	1 (14%)	2 (7%)	3 (9%)	
MG-ADL at enrollment, median (IQR)	2 (2–5)	2 (0–4.5)	2 (0–5)	0.501
**Details of tacrolimus medication**				
Age of starting tacrolimus, median (IQR), y	70 (69–77)	74 (70–80)	74 (69–79)	0.405
Duration of MG at tacrolimus initiation, median (IQR), m	2 (1–11)	11 (2.5–23)	9 (2–22)	0.200
Duration from starting tacrolimus to the last follow-up or withdrawal, median (IQR), m	28 (25–44)	40.5 (27–54)	36 (27–52)	0.454
Dose of tacrolimus at the final evaluation, median (IQR), mg/day	1.75 (1.0–2.5)	1.0 (1.0–1.5)	1.0 (1.0–2.0)	0.233
The last tacrolimus concentration, median (IQR), ng/ml	4.65 (3.1–7.2)	4.25 (2.7–5.0)	4.25 (2.85–5.7)	0.579
**Comorbidities**				
Without comorbidity	1 (14%)	1 (4%)	2 (6%)	0.275
Number of comorbidities, median (IQR)	2 (1.5–4)	2 (1–4)	2 (1–4)	0.220

*AChR, acetylcholine receptor; MG, Myasthenia Gravis; MGFA, Myasthenia Gravis Foundation of America; IQR, interquartile range; MG-ADL, MG Activities of Daily Living; IQR, interquartile range; EQ-5D-5L, five-level EuroQol five-dimensional questionnaire; MG-QOL-15R, revised 15-item Myasthenia Gravis Quality of Life scale; SSQ, simple single question*.

For the 13 patients (36%) who could not achieve adequate tacrolimus concentration by adjustment of dosage, WZC was prescribed. Among them, two tablets of WZC were administered together with tacrolimus every time, and the final dosage of tacrolimus was 1.5 (1–2) mg/day. The median tacrolimus concentration rose significantly (*p* = 0.012, paired *t*-test) from 2.75 (1.4–3.8) ng/ml (detected within 1 month before coadministration of WZC) to 5.95 (5.1–7.0) ng/ml (detected between the first and second months after coadministration of WZC), with an increase of 3.0 (2.4–4.5) ng/ml (Figure 2D). There were no patient-reported ADRs related to WZC.

The incidence of ADRs caused by tacrolimus was 23% (8/35) in the Tac group and 25% (9/36) in all the participants. Patient-reported ADRs included elevated blood sugar, higher blood pressure, itchy skin, lung infection, headache, palpitations, involuntary hand tremor, fluctuation of prostate-specific antigen, and gaining weight, all of which were not serious and eased by dosage adjustment and/or symptomatic treatment. One patient in the control group developed severe respiratory distress after taking tacrolimus for 1 week during hospitalization. Alleviation took place after withdrawal, and tacrolimus was not used again.

### Tacrolimus Without Corticosteroids vs. Tacrolimus With Corticosteroids in Treating VLOMG

Of the Tac group, 24 patients received tacrolimus without corticosteroids, and 12 coadministered corticosteroids (Table 2). The MGFA-PIS did not differ significantly between the two subgroups (Figure 2B). Remission was recorded in 46% of the non-corticosteroids group and in 36% of the corticosteroids group. The distributions of other statuses were as follows: improved [30% (non-corticosteroids) vs. 55% (corticosteroids)], unchanged [21% (non-corticosteroids) vs. 10% (corticosteroids)], and worsened [4% (non-corticosteroids) vs. 0% (corticosteroids)]. More relapse(s) or aggravation(s) during treatment occurred in patients receiving tacrolimus with corticosteroids. Notably, MGFA type III dominated the corticosteroids group (75%), while similar proportions of type II (33%) and type III (42%) were observed in the non-corticosteroids group (Figure 2A). There were no significant differences in other covariates, other measurements, ADRs, or tacrolimus discontinuation.

### Tacrolimus vs. Other Therapies in Treating VLOMG

Comparisons of covariates between the Tac group and the control group are summarized in Table 1. Concerning the 70 patients, the percentages of MGFA type differed significantly (*p* = 0.020). Type III dominated the Tac group (19/36, 53%), while type I appeared the most in the control group (15/34, 44%) and 53% (8/15) of these ocular MG patients required only single-agent therapy with cholinesterase inhibitors (Figure 2A). The Tac group used fewer corticosteroids [using: 11 (Tac) vs. 41% (control), discontinuation: 22 (Tac) vs. 12% (control), never use: 67 (Tac) vs. 47% (control), *p* = 0.015] and fewer other immunosuppressors [using: 6 (Tac) vs. 26% (control), discontinuation: 3 (Tac) vs. 6% (control), never use: 92 (Tac) vs. 68% (control), *p* = 0.038]. Fewer patients [6% (Tac) vs. 31% (control), *p* = 0.006] in the Tac group had digestive system diseases (including peptic ulcer, intestinal microflora disorders, gastric stromal tumor, severe indigestion, stomach polyps, and rectal polyps). No differences appeared in other clinical features or sociodemographic characteristics. Neither did covariates of the first visit subgroup.

Outcome measurements of the whole cohort and subgroups are listed in Table 3 without any significant differences. MG crisis developed in 4 patients (11%) of the Tac group and 3 (9%) of the control during follow-up. At the final evaluation, the MG-QOL-15R and SSQ scores of all the patients were 1 (0–6) and 8 (6–9), respectively; the EQ-5D-5L health value was 0.88 (0.77–1.00). The Supplementary Table 1 displays the results of multivariate analyses. No significant associations were found between tacrolimus administration and outcomes. In the whole cohort and the MGFA > I subgroup, corticosteroids administration was associated with more relapse(s) or aggravation(s) [whole: RR = 1.382 (95% CI, 1.052 to 1.815), *p* = 0.020. MGFA > I: RR = 1.566 (95% CI, 1.182–2.076), *p* = 0.002]. A higher MGFA type was highly correlated with a lower EQ-5D-5L health value in the whole cohort [β = −0.064 (95% CI, −0.124 to −0.004), *p* = 0.038]. Patients treated with plasmapheresis were more likely to obtain lower SSQ scores [β = −7.160 (95% CI, −11.209 to −3.112), *p* = 0.001] than those who never received plasmapheresis in the MGFA > I subgroup. A history of digestive system diseases was associated with lower SSQ scores and worse MGFA-PIS in all the patients [SSQ score: β = −2.328 (95% CI, −4.450 to −0.206), *p* = 0.032; MGFA–PIS: RR = 1.292 (95% CI, 1.073–1.556), *p* = 0.007]. Analyses of patients with MGFA > I revealed similar results [SSQ score: β = −2.917 (95% CI, −5.161 to −0.673), *p* = 0.012. MGFA-PIS: RR = 1.320 (95% CI, 1.089–1.600), *p* = 0.005]. In addition, those with digestive diseases in this subgroup reported more relapse(s) or aggravation(s) than those without such diseases [RR = 1.785 (95% CI, 1.015 to 3.139), *p* = 0.044].

According to patients' description at follow-up, 45 (67%) had no ADRs, and a total of 28 ADRs were reported, consisting of 9 [25% (9/36)] caused by tacrolimus, 7 [26% (7/27)] by corticosteroids, 6 [11% (6/52)] by cholinesterase inhibitors, 3 by other drugs (anticoagulant, antiplatelet, and statins), and 3 by unclear drugs (1 skin lesion, 1 muscle pain, and 1 gastrointestinal dysfunction). The incidence of ADRs related to different drugs showed no differences between the Tac group and the control group. ADRs caused by corticosteroids included Cushing syndrome, palpitations, cataracts, elevated blood sugar, femoral head diseases, impaired kidney function, and skin lesions. Cholinesterase inhibitors caused depression, excessive saliva production, palpitations, unexplained fever, gastrointestinal dysfunction, tremor, and paresthesia.

## Discussion

Tacrolimus was effective for VLOMG in this study. Eighty percent of the Tac group achieved remission (43%) or improved (37%) according to the MGFA-PIS. Outcome measurements indicated tacrolimus non-inferior to other medications, consistent with one study on MG of Osserman grades III and IV (16). Although the positive effects here seemed weaker than several earlier studies that reported remission rates ≥ 64% (11, 12, 15, 16, 21), the differences could be explained by the already achieved improvement before baseline and relatively stable MG status at enrollment. Almost all the previous studies started with tacrolimus initiation, while the majority of our patients began with a notable duration of treatment; conceivably, there was less space for a positive effect to be displayed. One randomized controlled trial failing to reveal positive results faced a similar situation where the average MG-ADL at entry was only 1.8 (29). We analyzed the first visit Tac subgroup to remove the intervention of treatments before baseline, where MG-ADL decreased significantly during follow-up. Advantages of tacrolimus in generalized MG could be further suggested regarding the higher MGFA type in the Tac group and the marked proportion of ocular MG patients treated with cholinesterase inhibitors alone in the control group. It also agreed with the clinical consensus that tacrolimus works well as an add-on therapy for those who were intolerant or did not respond well enough to corticosteroids. Moreover, high quality of life in VLOMG patients treated with tacrolimus was observed in this study, with MG-QOL-15R close to the remission patients and EQ-5D-5L health utility value matching MGFA type I (30, 31). Covariates and outcomes did not display statistical differences between tacrolimus with and without corticosteroids in this study. Nonetheless, the results should be interpreted carefully and conservatively according to the small sample size, since the effects of some non-statistically significant differences (such as MGFA type) could exist. Though it was insufficient to conclude that the two therapies held comparable efficacy, at least part of the VLOMG population in China could benefit from tacrolimus monotherapy, achieving relatively satisfying improvement or maintenance while avoiding the ADRs of steroids. Similar comparisons were rare except for one retrospective study of generalized MG showing coadministration with corticosteroids better (32). And it was not clear whether to use tacrolimus directly as a single drug, or to take corticosteroids first to stabilize and then tacrolimus alone for maintenance. Studies with larger samples and longer follow-up are required to evaluate the effectiveness of tacrolimus monotherapy. The only measurement showing differences was more relapse(s) or aggravation(s) in the corticosteroids group, which is reasonable since steroids are usually the first-line treatment for more severe MG (2). It was worth noting that the recurrence rate of the whole Tac group was relatively high. However, this could be explained from the following four aspects. First, the incidence of relapse(s) or aggravation(s) in the control group was similarly high, indicating reasons other than tacrolimus to account for this abnormity. Second, older age of onset was discovered to be a remarkable predictor for OMG generalization (33). Third, the definition of relapse(s) or aggravation(s) was not consistent across studies. Lower rates were reported in studies where relapse was defined as the reappearance of extraocular symptoms (33) and aggravation as increases of quantitative scores (13, 32), while we counted the recurrence of ocular symptoms and used more subjective assessment. Fourth, our patients' compliance was probably lower owing to COVID-19 pandemic where visiting doctors and getting prescription drugs were restricted. We found some patients discontinued their medications without physicians' orders. For the future researches and clinical practice, we suggest including ocular disorders in the criteria of relapse due to the deterioration of life quality; VLOMG patients should be followed up more closely and online mode is recommended. Although we identified no obvious difference in univariable comparisons of outcomes between patients with generalized and ocular MG, the two subgroups were unbalanced (30 patients vs. 5 patients) so the statistical power might be unconvincing to draw conclusions. Clinical characteristics and medication details did not appear to affect the effectiveness of tacrolimus, possibly because of the small sample size. Further investigations are needed to disclose the features of VLOMG patients who are more suitable to receive tacrolimus treatment. In short, considering the favorable results observed in our study and the devastating ADRs of corticosteroids in the elderly (23), tacrolimus without corticosteroids could be one of the treatment options for VLOMG.

For the VLOMG patients with unsatisfactory tacrolimus concentrations, coadministering WZC helped them reach the recommendation of 2 to 9 ng/ml with no obvious side effects (23). Other products of Wuweizi (Schisandra sphenanthera) were not used in this study, thus excluding the effect of non-WZC drugs on plasma levels. Active components of WZC compete with tacrolimus to bind metabolic enzymes (CYP3A5 and CYP3A4) and inhibit the *P*-glycoprotein-mediated efflux of tacrolimus, thereby, reducing the intestinal first-pass effect and remarkably increasing the concentration (17). Compared with increasing the dosage of tacrolimus, prescribing WZC did not merely achieve similar efficacy but provided extra profits of preventing hepatotoxicity and reducing the financial burden (19). Neither tacrolimus nor WZC is covered by basic medical insurance in China. Assuming 30 days a month and considering the prices of drugs used by our patients, taking tacrolimus at 3 mg/day costs 2,700 RMB (423.63 USD) per month, while tacrolimus 1.5 mg/day plus WZC 4 capsules/day costs only 1,470 RMB (230.64 USD). Notably, the average pension for retirees was estimated to be 3,500.60 RMB (549.24 USD) per month in 2021 according to the Ministry of Human Resources and Social Security of China, which means that elevating tacrolimus dosage could bring a heavy burden to VLOMG patients. Instead, usage of WZC economically benefits elderly patients who metabolize tacrolimus more quickly. Although the small sample size of WZC takers in this study limited the validity, our observations complemented the previous study in younger MG patients aged 36 (27–50) years (18).

In multivariable analyses, a history of digestive diseases was identified to be associated with worse MGFA-PIS and lower SSQ scores. Although earlier studies on MG rarely reported gastrointestinal diseases, these studies did not examine patients with VLOMG. While polyps, gastric stromal tumors, and indigestion showed no direct relevance to MG, a higher incidence of peptic ulcer in the control group indicated certain hazard factors. Corticosteroids were suggested given their contribution to ulcer development. In addition, dysregulated gut microbiota seemed to affect MG manifestations by promoting an imbalance in T cell populations (34). A higher MGFA type was found to be correlated with a lower EQ-5D-5L health value, in accordance with the foregoing findings (30). Plasmapheresis seemed to be correlated with a worse prognosis, reflecting more serious disease conditions (two with MGFA III, one with MGFA IV), which led to worse outcomes. The small sample size and lack of plasmapheresis in the Tac group limited further inference.

Most ADRs caused by tacrolimus in our study were mild and easily relieved, which has also been reported by the previous studies, while the incidence varied (10–13, 29). Weight gaining, an unreported ADR, could be explained by the diabetogenicity of calcineurin inhibitors (35). This also suggests blood glucose be more closely monitored and carefully balanced in VLOMG patients after taking tacrolimus, considering the higher prevalence of diabetes or prediabetes in the elderly and the well-recognized ADR of hyperglycemia. Fluctuation of prostate-specific antigen has not been reported, but sirolimus, which has a similar structure to that of tacrolimus, was found to be associated with a decrease of prostate-specific antigen in the kidney recipients (36). As expected, almost all the participants were affected by comorbidities, and higher incidences of chronic diseases were displayed (5, 7, 32). A total of 77% were taking concomitant drugs such as beta-blockers and statins, both of which were reported to worsen MG, although these reactions were not observed in our patients (37). As earlier studies showed that more than two comorbidities were related to poorer outcomes (8), the number of comorbidities in our patients was noteworthily 3 (2–4). One in the Tac group died of cerebral infarction, confirming the destructive impact of comorbidities burden. The occurrence of MG crisis during follow-up in our study was higher than the reported Chinese data which covered all the age groups (7). For the two dying of MG crisis in the control group, there seemed no direct associations with medications regarding the normal therapies they received. Failure to quickly identify the impending respiratory paralysis and complex comorbid conditions could explain the death of an outbreak at home; the other patient died from tracheotomy-related death together with MG crisis, unresponsive wakefulness syndrome, and pneumonia, all being hazard factors for tracheotomy complications (38). We need to focus more on the prevention of myasthenia crisis, which is typically precipitated by poor control of generalized MG. In summary, VLOMG patients demand conscientious and individualized management, and more specific treatment guidelines and consensus are needed.

This study has several limitations. First, this is a single-center retrospective observational study with relatively small sample size. More controlled studies and high-quality RCTs are necessary to confirm our findings. Second, follow-up was conducted through telephone interviews, and only patient-reported outcomes were adopted, which introduced more recall bias and reduced the objectivity of our study. However, the lack of face-to-face follow-up seemed acceptable under the COVID-19 pandemic, and patient-reported outcomes provided more comprehensive depictions of the treatment impact. For a more comprehensive evaluation, combining physician examination scales with patient-reported scales is a superior option. Third, the rate of contact loss was relatively high, which could cause the measured outcomes to be worse than the actual outcomes since patients with less severe symptoms tended not to keep in touch with their doctors. Conversely, lacking measurements of the deceased may lead to a better estimation of outcomes, as patients who died during follow-up were probably under poor conditions.

In conclusion, tacrolimus is effective and safe in the treatment of VLOMG. Our results also show that tacrolimus monotherapy without corticosteroids could be one choice for VLOMG initial and maintenance treatment, especially for those who cannot tolerate or do not want to use corticosteroids and other immunosuppressors. More studies are needed to confirm the efficacy of tacrolimus monotherapy and to select patients with VLOMG who are suitable for such treatment. Coadministering Wuzhi capsules can effectively and safely improve tacrolimus concentrations when needed. Patients with higher MGFA types or comorbidities need more frequent monitoring and cautious management.

## Data Availability Statement

The raw data supporting the conclusions of this article will be made available by the authors, without undue reservation.

## Ethics Statement

The studies involving human participants were reviewed and approved by Institutional Review Board and Ethics Committee at Peking University First Hospital. The patients/participants provided their written informed consent to participate in this study.

## Author Contributions

YZhe contributed with drafting and revising the manuscript, study concept and design, and acquisition of data. XY contributed with drafting and revising the manuscript, study design, acquisition of data, and statistical analysis. CZ, RL, HJ, HH, FL, YZha, and YY contributed with the acquisition of data. ZW contributed with revising the manuscript. FG contributed with revising the manuscript, study concept and design, and interpretation of the data. All authors contributed to the article and approved the submitted version.

## Funding

This study was supported by the Scientific Research Seed Fund of Peking University First Hospital (2018SF033). Funding bodies did not play a role in the collection, analysis, and interpretation of data. Funding bodies did not contribute to the writing of this manuscript.

## Conflict of Interest

The authors declare that the research was conducted in the absence of any commercial or financial relationships that could be construed as a potential conflict of interest.

## Publisher's Note

All claims expressed in this article are solely those of the authors and do not necessarily represent those of their affiliated organizations, or those of the publisher, the editors and the reviewers. Any product that may be evaluated in this article, or claim that may be made by its manufacturer, is not guaranteed or endorsed by the publisher.

## Abbreviations

MG: myasthenia gravis
VLOMG: very-late-onset MG
Tac: tacrolimus
ADRs: adverse drug reactions
MGFA: Myasthenia Gravis Foundation of America
MGFA-PIS: MGFA post-intervention status
MG-ADL: MG activities of daily living
AChR: acetylcholine receptor
WZC: Wuzhi capsules
IVIg: intravenous immunoglobulin
MG-QOL-15R: revised 15-item Myasthenia Gravis Quality of Life scale
SSQ: simple single question
EQ-5D-5L: five-level EuroQol five-dimensional questionnaire
IQR: interquartile range
RR: rate ratio
95%CI: 95% confidence intervals.
