# Supplementary material for: Efficacy and Safety of Tacrolimus Therapy for a Single Chinese Cohort With Very-Late-Onset Myasthenia Gravis
Source: Front Neurol. 2022 Mar 30;13:843523. doi: 10.3389/fneur.2022.843523 (PMC9007732; doi:10.3389/fneur.2022.843523)
Supplement: Supplementary file 1 [file Table_1.DOCX]

Supplementary Material

**Supplementary Table** Multivariate analyses of associations between tacrolimus administration and outcomes

a. Subtract MG-ADL score at enrollment from the score at the last follow-up; b. Assignment rules of this item: remission=1, improved=2, unchanged=3, worsened=4; c. Adjusted for confounding factors (variables differed significantly in univariable comparison) of corticosteroids usage, other immunosuppressor usage, MGFA type, and digestive system diseases; d. There’s no confounding factor needed to adjust; e. Adjusted for confounding factors of hypertension; f. Adjusted for confounding factors of corticosteroids usage, plasmapheresis usage, and digestive system diseases. MG-ADL, MG Activities of Daily Living; EQ-5D-5L, five-level EuroQol five-dimensional questionnaire; MG-QOL-15R, revised 15-item Myasthenia Gravis Quality of Life scale; SSQ, simple single question; MGFA, Myasthenia Gravis Foundation of America; MGFA-PIS, Myasthenia Gravis Foundation of America Post-Intervention Status; β, partial regression coefficient; CI, confidence interval; RR, rate ratio; Whole, 67 patients completing the last follow-up except 3 deceased; First visit, 19 patients who first visited our institution and were newly diagnosed with MG at enrollment; MGFA>1, 50 patients whose MGFA type was higher than I.

|  | | **Multiple linear regression** | | | | | | | | **Multiple modified Poisson regression** | | | |
| --- | --- | --- | --- | --- | --- | --- | --- | --- | --- | --- | --- | --- | --- |
| **Outcomes** | | **Change of MG-ADL^a^** | | **MG-QOL-15R** | | **SSQ** | | **EQ-5D-5L health value** | | **MGFA-PIS^b^** | | **Relapse (s) or aggravation (s)** | |
| **variables** | **patients** | β (95%CI) | p value | β (95%CI) | p value | β (95%CI) | p value | β (95%CI) | p value | RR (95%CI) | p value | RR (95%CI) | p value |
| **Tacrolimus** (Compared with no tacrolimus) | Whole^c^ | 0.170  (-1.440, 1.780) | 0.834 | 0.426  (-2.725, 3.577) | 0.788 | 0.239  (-1.300, 1.777) | 0.758 | -0.032  (-0.161, 0.096) | 0.614 | 1.084  (0.856, 1.372) | 0.505 | 0.842  (0.495, 1.432) | 0.526 |
|  | First visit^d^ | 0.778  (-1.690, 3.245) | 0.514 | -1.444  (-4.902, 2.013) | 0.389 | 0.778  (-1.642, 3.197) | 0.505 | 0.080  (-0.018,0.179) | 0.102 | 1.429  (0.977, 2.088) | 0.066 | 1.000  (0.261, 3.836) | 1.000 |
|  | MGFA>1^f^ | 0.055  (-2.198, 2.308) | 0.961 | 1.518  (-3.018, 6.053) | 0.503 | -0.819  (-2.711, 1.072) | 0.386 | -0.105  (-0.295, 0.085) | 0.273 | 1.118  (0.840, 1.489) | 0.444 | 0.904  (0.505, 1.617) | 0.733 |
| **Corticosteroids** (Compared with no corticosteroids) | Whole | 0.720  (-0.217, 1.657) | 0.130 | -1.439  (-3.273, 0.394) | 0.122 | 0.438  (-0.461, 1.337) | 0.334 | 0.071  (-0.004, 0.146) | 0.062 | 0.940  (0.836, 1.057) | 0.304 | **1.382**  **(1.052, 1.815)** | **0.020** |
|  | MGFA>1 | 0.852  (-0.322, 2.026) | 0.150 | -1.652  (-4.015, 0.712) | 0.166 | 0.470  (-0.517, 1.456) | 0.342 | 0.075  (-0.024, 0.174) | 0.135 | 0.928  (0.810, 1.063) | 0.282 | **1.566**  **(1.182, 2.076)** | **0.002** |
| **Digestive system diseases** (Compared with no digestive system diseases) | Whole | 0.708  (-1.381, 2.797) | 0.500 | 3.249  (-0.839, 7.336) | 0.117 | **-2.328**  **(-4.450, -0.206)** | **0.032** | -0.112  (-0.278, 0.055) | 0.184 | **1.451**  **(1.104, 1.908)** | **0.008** | 1.327  (0.751, 2.345) | 0.330 |
|  | MGFA>1 | 0.922  (-1.663, 3.507) | 0.476 | 4.276  (-0.927, 9.479) | 0.105 | **-2.917**  **(-5.161, -0.673)** | **0.012** | -0.177  (-0.395, 0.042) | 0.110 | **1.458**  **(1.091, 1.947)** | **0.011** | **1.785**  **(1.015, 3.139)** | **0.044** |
| **MGFA type** (Compared with MGFA type I) | Whole | -0.171  (-0.928, 0.587) | 0.654 | 1.217  (-0.266, 2.699) | 0.106 | -0.468  (-1.193, 0.257) | 0.201 | **-0.064**  **(-0.124, -0.004)** | **0.038** | 1.015  (0.890, 1.158) | 0.826 | 1.192  (0.950, 1.495) | 0.130 |
| **Plasmapheresis** (Compared with no plasmapheresis) | MGFA>1 | -3.485  (-7.607, 0.637) | 0.095 | 2.719  (-5.580, 11.017) | 0.512 | **-7.160**  **(-11.209, -3.112)** | **0.001** | -0.244  (-0.592, 0.104) | 0.164 | 1.216  (0.713, 2.074) | 0.474 | 0.659  (0.317, 1.392) | 0.274 |
